# Gustatory thresholds and obesity: a comparative study of five main tastes

**DOI:** 10.1186/s40795-025-01125-y

**Published:** 2025-07-21

**Authors:** Hamidreza Khalighi, Hamed Mortazavi, Fahimeh Anbari, Masoumeh Sadat Eftekhari, Nahid Mohammadnia, Homa Mirzaei, Sara Nashibi

**Affiliations:** 1 Oral Medicine Department, School of Dentistry, Shahid Beheshti University of Medical Sciences, Tehran, Iran; 2 School of Dentistry, Shahid Beheshti University of Medical Sciences, Tehran, Iran; 3 General Dental Practitioner, Tehran, Iran; 4 Dentofacial Deformities Research Center, Research Institute for Dental Sciences, Shahid Beheshti University of Medical Sciences, Tehran, Iran

**Keywords:** Taste perception, Taste threshold, Obesity, Overweight

## Abstract

**Background:**

Obesity is an important public health concern, which has dramatically grown in the last decades. Taste sensation determines food preferences and could contribute to obesity. Considering the conflicting results about the association of gustatory threshold and overweight/obesity status of individuals, this study designed to compare gustatory threshold of five main tastes (sweet, umami, salty, bitter, and sour) among individuals with overweight/obesity and with a normal BMI.

**Methods:**

In this case-control and population-based study, 100 adults participated, divided into two equal number of normal and overweight/obese groups. To measure gustatory threshold of sweet, umami, salty, bitter, and sour tastes, solutions of sucrose, monosodium glutamate, sodium chloride, quinine hydrochloride and citric acid were prepared respectively. Three millimeters of each solution was dropped on the right side of the posterior tongue, and the same volume of distilled water was dropped on the left side. The concentration at which the participant perceived the taste was considered the gustatory threshold. The data was analyzed in SPSS software version 22 with the Mann-Whitney test and Spearmen correlation coefficient.

**Results:**

No statistically significant differences were observed between the gustatory thresholds of the five main tastes among participants with normal BMI and with overweight/obesity. Moreover, among the numerical value of BMI and perceived concentrations of each of the tastes, no association was found (*p* value > 0.05).

**Conclusions:**

According to the findings obtained from this study, no significant statistical difference in gustatory threshold of five main tastes was observed among individuals with overweight/obesity and normal weight.

**Supplementary Information:**

The online version contains supplementary material available at 10.1186/s40795-025-01125-y.

## Background

Obesity is an important public health concern, declared by the World Health Organization (WHO) in 1997 [1]. Obesity has dramatically grown in the last decades. Recent studies emphasized the association of obesity with several diseases, which proves the necessity of its prevention and treatment [2]. Overweight is a result of imbalance between dietary energy intake and energy expenditure [3]. Body mass index (BMI) is one of the most applicable methods to evaluate obesity, using height and weight measurements. The people with a BMI between 25 and 29.9 and over 30 kg/m^2^, are classified as overweight and with the obesity, respectively [4].

Both genetic and environmental factors are contributed to the obesity [5]. In addition, taste perceptions are determinants of nutritional route in individuals and could affect eating behaviors, and subsequently, body mass [6]. The taste sensation is perceived through taste buds, which are mostly found in the tongue, palate, and throat [7]. This sense is the final step in determining whether food enters the digestive tract or not [8].

Taste perception might be altered in individuals with obesity. Evidence suggests that taste can be affected by obesity, as the tongue is a target organ by this disease [9]. Intriguingly, it has been shown that taste receptor expression found to be different in extra-oral organs e.g. hypothalamus, brain stem, and duodenum of lean and obese mice [10]. Despite growing literature in this area, much research on obesity still focuses primarily on eating behaviors, while less is known about sensory perception alterations in obesity [11].

Among the five basic tastes (i.e., sweet, salty, sour, bitter, and umami), sweet taste has been the most widely studied in relation to obesity [12]. Various studies have declared a higher sweet taste threshold in individuals with obesity. The decrease in the ability to recognize sweet taste by individuals with obesity might lead to more consumption of sweet foods, which could result in the occurrence of severe obesity and the nutritional status [13, 14]. However, findings regarding sweet taste threshold in obesity remain controversial and other studies found no significant differences or even increased sweet taste sensitivity [15].

Regarding other basic tastes, studies on salty, sour, and bitter taste perception in individuals with obesity have obtained diverse results. Most studies suggest that individuals with overweight or obesity present decreased sensitivity and increased thresholds compared to normal-weight individuals, while a few investigations reported no significance differences in these tastes [15]. These findings indicate a trend toward reduced taste sensitivity in obesity, but inconsistencies and methodological differences limit definitive conclusions.

The umami taste was first introduced nearly 110 years ago by Kikunae Ikeda. This taste could be distinguished in meat and fish and has perhaps evolved to encourage the consumption of high-protein foods [16]. The amino acid monosodium l-glutamate (MSG) is the main ingredient of umami taste and could be strongly enhanced by ribonucleotides such as guanosine monophosphate (GMP) and inosine monophosphate (IMP). The glutamate class G protein-coupled receptor (GPCR) dimer TAS1R1 + TAS1R3 is the main receptor, mediating the taste of umami [17]. A recent study revealed that umami taste is linked with healthy diets, though this taste has not been considered widely in obesity studies [18].

A 2023 systematic review of taste perception in obesity identified eight relevant studies; among these, six showed similar thresholds between BMI groups, three found higher thresholds in individuals with obesity for some tastes, and two reported higher thresholds for sweet, salty, bitter, and sour tastes in individuals without obesity [6]. These inconsistencies highlight the lack of consensus on how taste perception varies with BMI.

In summary, several studies have evaluated changes in gustatory thresholds across different BMI classifications. However, the umami taste has not been considered enough in these investigations. In addition, no consensus has been made regarding whether the taste perception alters in obesity or not.

According to the controversy on the findings of various research regarding the relationship of gustatory threshold and the people with overweight/obesity, as well as the lack of investigations which included the umami taste, the present study designed to evaluate gustatory threshold of five main tastes (sour, salty, sweet, bitter, and umami) among individuals with overweight/obesity and with a normal BMI.

## Methods

The study protocol was approved by the Institutional Ethics Committee, School of Dentistry, Shahid Beheshti University of Medical Sciences, Tehran, Iran, and the ethical approval code is: IR.SBMU.RIDS.REC.1395.353. Between September 2020 and July 2022, gustatory thresholds of 100 randomly selected adults (mean age: 26 ± 6.96), who presented to an academic center of dentistry were evaluated. All participants approved and signed the informed consent before their presence in the study. Sample size was evaluated via SIQR formula and PASS software. A questionnaire was developed for the present study to obtain demographic data and medical history of patients. Individuals with a history or presence of diseases mentioned in the questionnaire were excluded from the study in order to prevent the interference with the results of the experiment [19–25]. Moreover, a table was designed to collect the variables measured in the experiment (Supplementary Table S1, S2).

The height (meter) and weight (kg) of all individuals who entered the study were evaluated using the same device and subsequently, the BMI was calculated for each individual. The participants were classified into normal (BMI: 20–25 kg/m^2^) and people with overweight/obesity (BMI > 25 kg/m^2^) groups (half of the participants in each group). The distribution of age and gender variables was approximately equal between the two experimental groups.

To measure gustatory threshold of 5 main tastes (sweet, umami, salty, bitter, and sour), two days before the beginning of the experiment, solutions of sucrose, monosodium glutamate, sodium chloride, quinine hydrochloride and citric acid (Merck, Darmstadt, Germany) were prepared respectively and maintained in the refrigerator. Prior to beginning of the study, the temperature of solutions was reduced to reach the room temperature, following the standards for taste perception experiments [26]. The concentrations of solutions are shown in Table 1 [26–28].

**Table 1:** Concentration of the used solutions based on molar

Solution type Number of solution	Sucrose	Sodium chloride	Acid citric	Quinine hydrochloride	Monosodium glutamate
1	0.01	0.01	0.00032	0.0000032	0.09
2	0.032	0.032	0.001	0.00001	0.36
3	0.1	0.1	0.0032	0.000032	1.05
4	0.32	0.32	0.01	0.0001	-
5	1	1	0.032	0.00032	-

The survey started at 9.00 a.m, in a quiet and odour-free environment to avoid interpretation with taste sensation. The room environment was conditioned following the standards for taste perception examinations (23–24 _C; 40–50% relative humidity; light with standard solar light 15,000 lx) [26, 29]. The participant was asked not to eat, smoke, and use gum, and mouthwash for one hour before the visit. It was described to the participant to inform at the moment of taste sensation. After rinsing mouth with distilled water, 3 milliliters of the lowest concentration of solutions of a taste were placed on the right side of the posterior tongue with a dropper, in a random order. The same volume of distilled water, as a control stimulus, was dropped on the left side of posterior tongue to ensure that any taste sensation reported is due to the test solution [30, 31]. The concentration at which the participant recognized the taste was considered the gustatory threshold. Higher concentrations were used when the participant was unable to identify the taste to achieve correct recognition and determine the gustatory threshold. Between applying different concentrations, participants were given two minutes for rinsing their mouths with distilled water.

The results of the experiments were analyzed via SPSS software version 22. For comparison of significant differences between the group with normal BMI and the group with overweight/obesity, a Mann-Whitney analysis was performed for each taste. Moreover, the correlation between numerical values of BMI and gustatory threshold of each taste, Spearman rank correlation coefficient was reported. In addition, ordinal logistic regression was used to declare the effect of age, sex, and BMI on gustatory threshold for five main tastes. A significant difference was defined as a probability value less than 0.05.

## Results

The distribution of gustatory thresholds of five tested tastes among normal and people with overweight/obesity could be observed in Table 2. The highest frequency of gustatory thresholds for sour, salty, sweet, bitter, and umami tastes was 0.001, 0.032, 0.032, 0.0000032, and 0.009 molar, respectively.

**Table 2:** Frequency and percentage of concentrations of taste of five tastes perceived by the normal and overweight individuals

	BMI	The used concentrations					*P* value
		1	2	3	4	5	
salty	Normal	3(6%)	34(68%)	12(24%)	1(2%)	0	0.768
	overweight	2(4%)	37(74%)	11(22%)	0	0	
sweet	Normal	18(36%)	26(52%)	6(12%)	0	0	0.982
	overweight	18(36%)	26(52%)	5(10%)	1(2%)	0	
sour	Normal	11(22%)	24(48%)	13(26%)	2(4%)	0	0.309
	overweight	6(12%)	26(52%)	17(34%)	1(2%)	0	
bitter	Normal	35(70%)	8(16%)	7(14%)	0	0	0.941
	overweight	36(72%)	3(6%)	11(22%)	0	0	
umami	Normal	27(54%)	20(40%)	3(6%)	0	0	0.565
	overweight	32(64%)	14(28%)	4(8%)	0	0	

According to the Mann-Whitney test, statistically significant differences were not observed among the gustatory thresholds of five main tastes between participants with normal BMI and with overweight/obesity (*p* value > 0.05).

According to Spearman rank correlation coefficient, in assessment among numerical values of BMI and perceived concentrations of each of the tastes, no association was found among the net BMI score and perceived concentrations of sodium chloride (*p* = 0.62), sucrose (*p* = 0.67), acid citric (*p* = 0.54), quinine hydrochloride (*p* = 0.78), and monosodium glutamate (*p* = 0.11). Furthermore, the logistic regression model showed that gustatory threshold was not affected by age and gender.

## Discussion

The present study examined the difference in perception of five main tastes among two groups of individuals with normal weight and overweight/obesity. In assessment of gustatory perception of five main tastes (sweet, umami, salty, bitter, and sour), no statically significant association was found between individual’s BMI and gustatory threshold. Several studies have performed research to find out the correlation between the individual’s BMI and gustatory threshold, however controversial findings have been made. Recent studies accomplished comprehensive reviews on the studies that investigated taste perception among people with different stages of body mass index. The results showed contradictory relationships between the performed studies that could be a result of different methods, different target population, and the presence of various influencing factors [6, 15].

Assessment of sweet taste perception has always been a subject of interest, according to the indispensable role of carbohydrates consumption in obesity. Various studies have pointed out a greater gustatory threshold for sweet taste in individuals with obesity [13, 32, 33]. However, there is no definitive relationship between gustatory thresholds and obesity, due to contradictory results. In 2020, the study by J. Leohr et al. demonstrated that the perception of sweetness is similar between subjects with/without obesity, which is in line with the results of the current study [34]. However, E. Martinez-Cordero reported lower perception threshold for sweet taste in females with overweight, in comparison with normal weight [35]. Various studies presented that sweet taste perception depends on gender. In 2015 A. Deglaire et al. demonstrated greater fat and salt preferences in both men/women with obesity, while a higher liking score for sweet foods with additional sugar only in women with obesity [36]. Moreover in 2018 A. W. B. van Langeveld et al. concluded a higher consumption of foods with fat, umami and salt tastes in individuals with obesity in a Dutch community and a preference for sweet-containing foods in women [37]. Furthermore, the study by E. L. Feeney et al. in 2017 found a lower sweet taste intensity in men with overweight/obesity, while no correlation was detected among women with normal weight and overweight/obesity in Irish school children [38]. Additionally, a study by J. Y. Low wt al. in 2016 evaluated the sweet taste function via measuring sweetness intensity, and thresholds of sweet taste detection/recognition. This study has not obtained any significant differences in the function of sweet taste among the participants with normal weight and overweight/obesity and found that sweet taste might be associated with other variables such as food consumption rather than anthropometric properties [39]. These findings indicate the important role of other influencing factors on sweet gustatory threshold.

The umami taste makes foods more palatable and umami substances trigger salivary secretion and increase appetite. A robust association has been observed between obesity and umami taste [12, 40]. Several studies investigated the gustatory threshold for umami taste in individuals with overweight/obesity. The study by J. Overberg et al. reported higher umami, salty, and bitter thresholds in adolescents and children with obesity, compared to those with normal BMI [41]. However, the study by S. Y. Lim et al. found no relationship between umami taste threshold with individual BMI, while body composition was correlated with umami taste perception [42]. The findings of the current study observed no correlation between umami taste recognition threshold and individual BMI, which is in line with previous studies [43, 44]. Various variables might influence the BMI relationship and umami taste threshold, which has led to different observations among related studies.

Salt consumption might affect body weight via increasing in liquid absorption. High gustatory threshold for salty taste might led to enhanced salt consumption [45]. A study has been conducted by D. C. Park, J et al. on detection thresholds of salty, sweet, sour, and bitter tastes among adults with normal weight and obesity. A significant difference was only observed for salty taste [46]. However, the study by U. Simchen et al. reported a lower bitter and sour taste perception in participants with normal weight, in comparison with those with overweight/obesity, while for sweet and salty tastes no differences were found, which is in line with the findings of present research. Additionally, different concentrations of quinine hydrochloride and acid citric used for bitter and sour tastes might be the reason of inconsistent results with current study findings [47].

Lower gustatory threshold of bitter taste might be associated with avoiding from eating specific foods [48]. Reduced intake of healthy, antioxidant-rich, and bitter-tasty foods results in obesity [49]. Therefore, studying bitter taste perception is important, according to its relation to food choices and feeding behaviors. The study by B. J. Tepper et al. reported a converse relationship between the perception of bitter taste and individual’s BMI and/or energy consumption [50]. Moreover, S. E. Choi et al. showed that better perception of bitterness in individuals is accompanied by lower energy consumption and a lower BMI [51]. However, in line with the present study, E. Martinez-Cordero at al. revealed no correlation between perception of bitterness and energy consumption or anthropometric variables [35]. In contrast, the study by U. Simchen et al. observed a lower bitter and sour taste perception in individuals with normal weight [47]. It seems that findings about bitterness perception and its relationship with anthropometrics are contradictory. Furthermore, gender, age, ethnicity, and genetics appear to influence perception of bitter taste [52].

Limited studies explored the correlation between sour taste recognition and obesity. The current study found no relationship between sour taste recognition threshold and obesity/overweight status of individuals. Various studies reported similar sour taste recognition among adults with/without obesity, which is in accordance with the results of current research [23, 35, 41]. In contrast, the study by C. Proserpio et al. presented higher threshold for sour taste in individuals with obesity, in comparison with normal weight controls [53]. In a more comprehensive view, sour stimuli perception by brain depends on age, sex, and condition. Considering the role of sour taste for signaling toxic substances, obesity might affect sour taste processing in the long term and be associated with health concerns [12].

The inconsistency between the results of the present study and previous literature might be attributed to differences in methodologies and target populations of the studies. The methods used to evaluate gustatory thresholds vary widely between studies, including differences in solution types, concentration ranges, delivery techniques (e.g., whole mouth, localized application, etc.), different methods of functioning of tastes (e.g., detection threshold measurement, recognition threshold measurement, suprathreshold measurement, etc.). These methodological variabilities might contribute to inconsistent findings across literature. Moreover, variances in demographic features of included participants such as, age, gender, and ethnicity may influence the taste perception and could be considered as a confounding factor in taste functioning research. Previous studies have presented that taste perception differs by gender [36, 38, 54]. Moreover, it has been indicated that body composition might be a more relevant factor to altered taste perception for obesity than BMI [39, 42].

In addition, external factors, including salivary flow, use of medications, and oral microbial composition may further complicate the relationship between obesity and gustatory thresholds [20, 22].

Considering the large heterogeneity in study protocols and populations, a systematic review or meta-analysis with subgroup analyses based on methodology, population characteristics, and confounding factors would be helpful in illuminating whether a relationship exists between obesity and taste perception.

### Limitations

This study assessed only taste recognition thresholds and did not include other measures of taste function, such as detection thresholds or suprathreshold intensity ratings. As these methods provide complementary information and are not interchangeable, future research should incorporate multiple approaches to more comprehensively evaluate taste perception in relation to obesity. In the present study, we evaluated taste perception at the posterior tongue region, however, whole-mouth methods may better reflect real-world tasting experiences. Future studies would benefit from combining both techniques to explore local vs. whole mouth taste perception in obesity. Moreover, smoking was not used as an exclusion criterion to avoid reducing participant numbers and to prevent discomfort or reluctance in disclosing smoking status. We acknowledge this as a limitation of the study.

## Conclusions

According to the findings obtained from this study, no significant statistical difference of gustatory threshold of five main tastes (sweet, umami, salty, bitter, and sour) was observed among individuals with overweight/obesity and normal weight. Given the conflicting results of similar studies on the relationship between gustatory threshold and overweight/obesity status of individuals, future research should focus on evaluating different influential variables in this regard.

## Electronic supplementary material

Below is the link to the electronic supplementary material.

**Supplementary Material 1**: **Supplementary table S1**: Patient information and medical history questionnaire, **Supplementary table S2**: Clinical assessment table

## Data Availability

Data is provided within the manuscript.

## References

[1] Haththotuwa RN, Wijeyaratne CN, Senarath U. Chap. 1 - Worldwide epidemic of obesity. In: Obesity and Obstetrics (Second Edition). edn. Edited by Mahmood TA, Arulkumaran S, Chervenak FA: Elsevier; 2020:3–8.

[2] Blüher M. Obesity: global epidemiology and pathogenesis. Nat Reviews Endocrinol. 2019;15(5):288–98. 10.1038/s41574-019-0176-8 30814686

[3] Moschonis G, Trakman GL. Overweight and obesity: the interplay of eating habits and physical activity. Nutrients 2023, 15(13). 10.3390/nu15132896 PMC10343820 37447222

[4] Khanna D, Peltzer C, Kahar P, Parmar MS. Body mass index (BMI): A screening tool analysis. Cureus. 2022;14(2):e22119. 35308730 10.7759/cureus.22119 PMC8920809

[5] Albuquerque D, Nóbrega C, Manco L, Padez C. The contribution of genetics and environment to obesity. Br Med Bull. 2017;123(1):159–73. 28910990 10.1093/bmb/ldx022

[6] Peinado BRR, Frazão DR, Bittencourt LO, Souza-Rodrigues RDd, Vidigal MTC, da Silva DT, Paranhos LR, Magno MB, Fagundes NCF, Maia LC. Is obesity associated with taste alterations? A systematic review. Front Endocrinol. 2023;14:1167119. 10.3389/fendo.2023.1167119 PMC10273260 37334283

[7] Wu B, Eldeghaidy S, Ayed C, Fisk ID, Hewson L, Liu Y. Mechanisms of Umami taste perception: from molecular level to brain imaging. Crit Rev Food Sci Nutr. 2022;62(25):7015–24. 33998842 10.1080/10408398.2021.1909532

[8] Nuemket N, Yasui N, Kusakabe Y, Nomura Y, Atsumi N, Akiyama S, Nango E, Kato Y, Kaneko MK, Takagi J. Structural basis for perception of diverse chemical substances by T1r taste receptors. Nat Commun. 2017;8(1):15530. 28534491 10.1038/ncomms15530 PMC5457512

[9] Rohde K, Schamarek I, Blüher M. Consequences of obesity on the sense of taste: taste buds as treatment targets? Diabetes Metabolism J. 2020;44(4):509–28. 10.4093/dmj.2020.0058 PMC7453985 32431111

[10] Herrera Moro Chao D, Argmann C, Van Eijk M, Boot R, Ottenhoff R, Van Roomen C, Foppen E, Siljee J, Unmehopa U, Kalsbeek A. Impact of obesity on taste receptor expression in extra-oral tissues: emphasis on hypothalamus and brainstem. Sci Rep. 2016;6(1):29094. 27388805 10.1038/srep29094 PMC4937374

[11] Tucker RM, Edlinger C, Craig BA, Mattes RD. Associations between BMI and fat taste sensitivity in humans. Chem Senses. 2014;39(4):349–57. 24591531 10.1093/chemse/bju006 PMC3982908

[12] Kure Liu C, Joseph PV, Feldman DE, Kroll DS, Burns JA, Manza P, Volkow ND, Wang G-J. Brain imaging of taste perception in obesity: a review. Curr Nutr Rep. 2019;8:108–19. 30945140 10.1007/s13668-019-0269-y PMC6486899

[13] Ribeiro G, Oliveira-Maia AJ. Sweet taste and obesity. Eur J Intern Med. 2021;92:3–10. 33593659 10.1016/j.ejim.2021.01.023 PMC8486680

[14] Low YQ, Lacy K, Keast R. The role of sweet taste in satiation and satiety. Nutrients. 2014;6(9):3431–50. 25184369 10.3390/nu6093431 PMC4179169

[15] Fathi M, Javid AZ, Mansoori A. Effects of weight change on taste function; a systematic review. Nutr J. 2023;22(1):22. 37158889 10.1186/s12937-023-00850-z PMC10165840

[16] Yoshida R, Ninomiya Y. Umami and MSG. Umami: taste for health. edn.: Springer International Publishing Cham; 2023. pp. 7–42.

[17] Diepeveen J, Moerdijk-Poortvliet TC, van der Leij FR. Molecular insights into human taste perception and Umami tastants: A review. J Food Sci. 2022;87(4):1449–65. 35301715 10.1111/1750-3841.16101 PMC9314127

[18] Fluitman KS, Hesp AC, Kaihatu RF, Nieuwdorp M, Keijser BJ, IJzerman RG, Visser M. Poor taste and smell are associated with poor appetite, macronutrient intake, and dietary quality but not with undernutrition in older adults. J Nutr. 2021;151(3):605–14. 33561272 10.1093/jn/nxaa400 PMC7948202

[19] Yang Y, Zhang C, Xiong T. Association between olfactory dysfunction and gustatory dysfunction: evidence from the National health and nutrition examination survey. Front Public Health. 2025;13:1519290. 40017542 10.3389/fpubh.2025.1519290 PMC11864946

[20] Pedersen AML, Sørensen CE, Proctor GB, Carpenter GH. Salivary functions in mastication, taste and textural perception, swallowing and initial digestion. Oral Dis. 2018;24(8):1399–416. 29645367 10.1111/odi.12867

[21] Costanzo A, Settapramote N, Utama-ang N, Wanich U, Lewin S, Keast R. Carbohydrate taste is associated with food intake and body mass in healthy Australian adults. Nutrients. 2021;13(11):3844. 34836099 10.3390/nu13113844 PMC8619819

[22] Mameli C, Cattaneo C, Panelli S, Comandatore F, Sangiorgio A, Bedogni G, Bandi C, Zuccotti G, Pagliarini E. Taste perception and oral microbiota are associated with obesity in children and adolescents. PLoS ONE. 2019;14(9):e0221656. 31509575 10.1371/journal.pone.0221656 PMC6738620

[23] Hardikar S, Höchenberger R, Villringer A, Ohla K. Higher sensitivity to sweet and salty taste in obese compared to lean individuals. Appetite. 2017;111:158–65. 27988366 10.1016/j.appet.2016.12.017

[24] Daly BP, Daly MP, Minniti N, Daly JM. Sense of Taste (Effect on Behavior). In: Encyclopedia of Human Behavior (Second Edition). edn. Edited by Ramachandran VS. San Diego: Academic Press; 2012: 373–378.

[25] Murtaza B, Hichami A, Khan AS, Ghiringhelli F, Khan NA. Alteration in taste perception in cancer: causes and strategies of treatment. Front Physiol. 2017;8:134. 28337150 10.3389/fphys.2017.00134 PMC5340755

[26] Melis M, Mastinu M, Naciri LC, Muroni P, Tomassini Barbarossa I. Associations between sweet taste sensitivity and polymorphisms (SNPs) in the TAS1R2 and TAS1R3 genes, gender, PROP taster status, and density of fungiform papillae in a genetically homogeneous Sardinian cohort. Nutrients. 2022;14(22):4903. 36432589 10.3390/nu14224903 PMC9696868

[27] Navabi N, Farzad M, ALAEI A. Taste threshold of four main tastes between healthy and diabetic individuals. 2009.

[28] Pepino MY, Finkbeiner S, Beauchamp GK, Mennella JA. Obese women have lower monosodium glutamate taste sensitivity and prefer higher concentrations than do normal-weight women. Obesity. 2010;18(5):959–65. 20075854 10.1038/oby.2009.493 PMC2987588

[29] Raithatha C, Bendini A, Garcia JA, Gasperi F, Gredilla E, Guerrero L, Insausti K, Malagoli M, Moya AS, del Castillo RR. Guidelines for Sensory Analysis of Protected Designation of Origin Food Products and Wines. 2021.

[30] Joseph PV, Mennella JA, Cowart BJ, Pepino MY. Psychophysical tracking method to assess taste detection thresholds in children, adolescents, and adults: the taste detection threshold (TDT) test. J Visualized Experiments: JoVE 2021(170):10.3791/62384 10.3791/62384 PMC8560331 33970129

[31] Brightman VJ. Chronic oral sensory disorders-pain and dysgeusia. In: Burket’s Oral Medicine: Diagnosis and Treatment. 8 edn. Edited by Lynch MA. Philadelphia: Lippincott Williams and Wilkins; 1984: 589.

[32] Gaesser GA. Carbohydrate quantity and quality in relation to body mass index. J Am Diet Assoc. 2007;107(10):1768–80. 17904937 10.1016/j.jada.2007.07.011

[33] Jilani H, Intemann T, Buchecker K, Charalambos H, Gianfagna F, De Henauw S, Lauria F, Molnar D, Moreno LA, Lissner L. Correlates of bitter, sweet, salty and Umami taste sensitivity in European children: role of sex, age and weight status-The IDEFICS study. Appetite. 2022;175:106088. 35597371 10.1016/j.appet.2022.106088

[34] Leohr J, Kjellsson MC. Sweet/Fat preference taste in subjects who are lean, obese and very obese. Pharm Res. 2020;37(12):244. 33215233 10.1007/s11095-020-02968-9 PMC7677291

[35] Martinez-Cordero E, Malacara-Hernandez JM, Martinez-Cordero C. Taste perception in normal and overweight Mexican adults. Appetite. 2015;89:192–5. 25681653 10.1016/j.appet.2015.02.015

[36] Deglaire A, Méjean C, Castetbon K, Kesse-Guyot E, Hercberg S, Schlich P. Associations between weight status and liking scores for sweet, salt and fat according to the gender in adults (The Nutrinet-Santé study). Eur J Clin Nutr. 2015;69(1):40–6. 25074389 10.1038/ejcn.2014.139

[37] van Langeveld AWB, Teo PS, de Vries JHM, Feskens EJM, de Graaf C, Mars M. Dietary taste patterns by sex and weight status in the Netherlands. Br J Nutr. 2018;119(10):1195–206. 29759103 10.1017/S0007114518000715

[38] Feeney EL, O’Brien SA, Scannell AG, Markey A, Gibney ER. Suprathreshold measures of taste perception in children - Association with dietary quality and body weight. Appetite. 2017;113:116–23. 28235619 10.1016/j.appet.2017.02.026

[39] Low JY, Lacy KE, McBride R, Keast RS. The association between sweet taste function, anthropometry, and dietary intake in adults. Nutrients. 2016;8(4):241. 27120614 10.3390/nu8040241 PMC4848709

[40] Stańska K, Krzeski A. The Umami taste: from discovery to clinical use. Pol J Otolaryngol. 2016;70(4):10–5. 10.5604/00306657.1199991 27387211

[41] Overberg J, Hummel T, Krude H, Wiegand S. Differences in taste sensitivity between obese and non-obese children and adolescents. Arch Dis Child. 2012;97(12):1048–52. 22995095 10.1136/archdischild-2011-301189

[42] Lim SY, Rosmawati D, Yatiman NH, Wong JE, Haron H, Poh BK. Umami detection threshold among children of different ethnicities and its correlation with various indices of obesity and blood pressure. Curr Res Food Sci. 2022;5:2204–10. 36387604 10.1016/j.crfs.2022.11.006 PMC9663310

[43] Bobowski NK, Mennella JA. Disruption in the relationship between blood pressure and salty taste thresholds among overweight and obese children. J Acad Nutr Dietetics. 2015;115(8):1272–82. 10.1016/j.jand.2015.02.017 PMC4516697 25843808

[44] Thu Hien VT, Thi Lam N, Cong Khan N, Wakita A, Yamamoto S. Monosodium glutamate is not associated with overweight in Vietnamese adults. Public Health Nutr. 2013;16(5):922–7. 22894833 10.1017/S1368980012003552 PMC10271504

[45] Zhou L, Stamler J, Chan Q, Van Horn L, Daviglus ML, Dyer AR, Miura K, Okuda N, Wu Y, Ueshima H. Salt intake and prevalence of overweight/obesity in japan, china, the united kingdom, and the united states: the INTERMAP study. Am J Clin Nutr. 2019;110(1):34–40. 31111867 10.1093/ajcn/nqz067 PMC6599742

[46] Park DC, Yeo JH, Ryu IY, Kim SH, Jung J, Yeo SG. Differences in taste detection thresholds between normal-weight and obese young adults. Acta Otolaryngol. 2015;135(5):478–83. 25739740 10.3109/00016489.2014.975370

[47] Simchen U, Koebnick C, Hoyer S, Issanchou S, Zunft H-J. Odour and taste sensitivity is associated with body weight and extent of misreporting of body weight. Eur J Clin Nutr. 2006;60(6):698–705. 16435003 10.1038/sj.ejcn.1602371

[48] Riccio MP, Franco C, Negri R, Ferrentino RI, Maresca R, D’alterio E, Greco L, Bravaccio C. Is food refusal in autistic children related to TAS2R38 genotype? Autism Res. 2018;11(3):531–8. 29282878 10.1002/aur.1912

[49] Turner A, Veysey M, Keely S, Scarlett C, Lucock M, Beckett EL. Interactions between bitter taste, diet and dysbiosis: consequences for appetite and obesity. Nutrients. 2018;10(10):1336. 30241292 10.3390/nu10101336 PMC6213475

[50] Tepper BJ, Banni S, Melis M, Crnjar R, Tomassini Barbarossa I. Genetic sensitivity to the bitter taste of 6-n-propylthiouracil (PROP) and its association with physiological mechanisms controlling body mass index (BMI). Nutrients. 2014;6(9):3363–81. 25166026 10.3390/nu6093363 PMC4179166

[51] Choi SE, Chan J. Relationship of 6-n-propylthiouracil taste intensity and Chili pepper use with body mass index, energy intake, and fat intake within an ethnically diverse population. J Acad Nutr Dietetics. 2015;115(3):389–96. 10.1016/j.jand.2014.09.001 25441957

[52] Shizukuda S, Marchini JS, Adell A, Santos MA, Brandao CFC, Lima CMM, Cunha SFC, Itikawa EN, Silvah JH. Influences of weight, age, gender, genetics, diseases, and ethnicity on bitterness perception: A narrative review of current methodological aspects. Nutrire. 2018;43:1–9.

[53] Proserpio C, Laureati M, Bertoli S, Battezzati A, Pagliarini E. Determinants of obesity in Italian adults: the role of taste sensitivity, food liking, and food neophobia. Chem Senses. 2016;41(2):169–76. 26671250 10.1093/chemse/bjv072

[54] van Langeveld AW, Teo PS, de Vries JH, Feskens EJ, de Graaf C, Mars M. Dietary taste patterns by sex and weight status in the Netherlands. Br J Nutr. 2018;119(10):1195–206. 29759103 10.1017/S0007114518000715

