# Supplementary material for: Gustatory thresholds and obesity: a comparative study of five main tastes
Source: BMC Nutr. 2025 Jul 21;11:142. doi: 10.1186/s40795-025-01125-y (PMC12281801; doi:10.1186/s40795-025-01125-y)
Supplement: Supplementary file 1 — Supplementary Material 1: Supplementary table S1: Patient information and medical history questionnaire, Supplementary table S2: Clinical assessment table [file 40795_2025_1125_MOESM1_ESM.docx]

| **Patient information and medical history** | | |
| --- | --- | --- |
| **Category** | **Variables** | **Answer** |
| **Demographics** | Patient Number |  |
|  | Age |  |
|  | Gender |  |
| **Medical History** | Olfactory dysfunction |  |
|  | Tate disorders |  |
|  | Neurologic disorders |  |
|  | Salivary gland diseases |  |
|  | Chronic use of medications |  |
|  | Lactation or pregnancy |  |
|  | Metabolic diseases |  |
|  | Chemotherapy and/or radiotherapy treatments |  |

Figure S 1. Patient information and medical history questionnaire

Figure S 2. Clinical assessment table

| **Clinical assessments** | | |
| --- | --- | --- |
| **Category** | **Variables** | **Answer** |
| **Anthropometric Data** | Weight |  |
|  | Height |  |
|  | Body Mass Index (BMI) |  |
| **Gustatory Thresholds** | Sweet |  |
|  | Umami |  |
|  | Salty |  |
|  | Bitter |  |
|  | Sour |  |
